# Revisiting 3D chromatin architecture in cancer development and progression

**DOI:** 10.1093/nar/gkaa747

**Published:** 2020-09-17

**Authors:** Yuliang Feng, Siim Pauklin

**Affiliations:** Botnar Research Centre, Nuffield Department of Orthopaedics, Rheumatology and Musculoskeletal Sciences, Old Road, University of Oxford, Oxford OX3 7LD, U.K; Botnar Research Centre, Nuffield Department of Orthopaedics, Rheumatology and Musculoskeletal Sciences, Old Road, University of Oxford, Oxford OX3 7LD, U.K

## Abstract

Cancer development and progression are demarcated by transcriptional dysregulation, which is largely attributed to aberrant chromatin architecture. Recent transformative technologies have enabled researchers to examine the genome organization at an unprecedented dimension and precision. In particular, increasing evidence supports the essential roles of 3D chromatin architecture in transcriptional homeostasis and proposes its alterations as prominent causes of human cancer. In this article, we will discuss the recent findings on enhancers, enhancer–promoter interaction, chromatin topology, phase separation and explore their potential mechanisms in shaping transcriptional dysregulation in cancer progression. In addition, we will propose our views on how to employ state-of-the-art technologies to decode the unanswered questions in this field. Overall, this article motivates the study of 3D chromatin architecture in cancer, which allows for a better understanding of its pathogenesis and develop novel approaches for diagnosis and treatment of cancer.

## INTRODUCTION

Precise spatiotemporal regulation of gene transcription is essential for normal cell function while transcriptional dysregulation is recognized as a universal feature of cancers. This fundamental process has a direct or indirect impact on virtually all other cancer hallmarks that include sustaining proliferative signaling, evading growth suppressors, resisting cell death, enabling replicative immortality, inducing angiogenesis, activating invasion and metastasis, reprogramming of energy metabolism, tumour microenvironment, inflammation, evading immune destruction and genome instability (1). Uncovering the molecular mechanisms of transcriptional dysregulation in cancer will provide novel insights into cancer development and progression and identify potential therapeutic targets. In a cell, transcriptional output is largely orchestrated by regulatory DNA elements, in particular enhancers. Dysregulated enhancers have been implicated in various cancers (2). In most cases, enhancers control gene expression through interaction with transcription factors (co-factors) and physically contact promoters through long-range interactions. In addition, enhancer–promoter interactions are framed by 3D genome architecture, such as topologically associated domains (TADs), which further aggregate into A/B compartments (3). Altered chromatin topology has been linked to aberrant enhancer–promoter interaction, which drives transcriptional activation of oncogenes and formation of cancer phenotype (4). This has led to current wave of studies to investigate how 3D genome architecture regulates chromatin biology in cancer. Despite this, the mechanisms adopted by cancer cells to construct their unique chromatin architectures remain relatively obscure. The cell nucleus contains a mixture of macromolecules with chromatin. Although it is crowed, sophisticated and precise gene regulation take place in this environment for cellular homeostasis in normal cells. This can be achieved via biomolecular condensates (phase-separated bodies) that spatiotemporally concentrate the biomolecules, altering their localization and activities in cells. The phase separation mechanism helps explain how chromatin is organized in the nucleus and implies that changes of this process may be involved in altering chromatin architecture for carcinogenesis. In addition, several new regulatory elements have been uncovered in the past few years, and their function in normal cells and cancer cells is beginning to emerge. Recent technological advances and new methods have expanded the options for analysing many samples simultaneously and in unprecedented depth, including at the single cell level. These recent advances help to tackle intriguing fundamental questions relating 3D genome organization: (i) Is enhancer–promoter interaction a driver or a passenger for gene activation? (2) What is the role of chromatin topology in transcriptional activation by enhancer–promoter interaction? (3) Does phase separation enable organization of chromatin topology in the nucleus? (4) How to capture multiplex chromatin interactions at the single-molecule level? (5) Is chromatin topology pre-established prior to transcriptional changes during cancer development?

In this review, we provide an overview of emerging chromatin regulatory elements relevant for cancer and the state-of-the-art approaches to measure the regulatory elements in bulk or single cell level, as well as their applications in cancer research. We will discuss whether the relation between enhancer–promoter interaction and transcription output is causal or correlative and examine previously unrecognized players regulating enhancer-promoter interactions. Moreover, we explore how 3D chromatin topology is altered in carcinogenesis and lead to aberrant enhancer-promoter interactions and tumorigenesis. We describe phase separation in chromatin topology with a focus on the potential roles of chromatin RNA, and the 3D genome mapping technology highlighting the necessity of using newly developed single-molecule approaches for understanding cellular heterogeneity, cancer stem cells, clonal evolution and therapeutic resistance in cancer research. Finally, we propose that identification of pre-established chromatin topology in the precancerous stage might be used as a novel approach for early warning or intervention of cancer.

## EMERGING REGULATORY ELEMENTS WITH ENHANCER FUNCTION IN CANCER

Aberrant enhancer activity orchestrates the dysregulated transcription program that dictate cancer development and progression. The enhancer landscape of tumour cells is extensively rewired as compared to their normal counterparts. Structural variations (e.g. inversions (5), translocations (6) and copy number alterations (7)) can tether enhancers close to the promoters of oncogenes and thereby drive their expression. For instance, increased copy number of the enhancer 650 kb centromeric to androgen receptor (AR) gene is the key driver for aberrant activation of AR, and drives the metastasis of prostate cancer (8). Apart from canonical enhancers, other non-coding elements are emerging as non-canonical enhancers implicated for cancer development.

### Enhancer-like promoter

H3K4me3 is a predominant histone mark for promoters, while H3K4me1 is a predominant histone mark for enhancers (9). The ratio of H3K4me3/H3K4me1 signal can be used as a quantitative measurement to assess the likelihood that a given regulatory element is a promoter or enhancer (i.e. a low ratio suggests enhancer-like while a high ratio suggests promoter-like element (10,11)). In breast cancer and leukemia cell lines, cloning enhancer-like and promoter-like transcription start sites (TSSs) fragments into downstream and upstream of a luciferase reporter gene has shown that enhancer-like TSSs can augment the transcriptional activity of promoter-like TSSs, while showing lower transcriptional activity than the promoter-like TSSs. This suggests that enhancer-like TSSs may function also as enhancers, in addition to being the sites of transcriptional initiation. Currently, Massively Parallel Reporter Assay (MPRA) (12) and Self-Transcribing Active Regulatory Region sequencing (STARR-seq) (13) are two most common genome-wide approaches to measure the enhancer activity. For instance, to measure genome-wide the enhancer activity of promoters, Salvatore Spicuglia lab performed STARR-seq in cervical cancer and leukemia cells and found that 3% of promoters displayed enhancer activity. In addition, they revealed that the activity of those enhancer-like promoters did not correlate with the transcription of their proximal genes, but instead, they enhanced the expression of distal genes. How those enhancer-like promoters regulated distal but not proximal gene expression needs further studies? In addition, Bing Ren lab developed an approach called cis-regulatory element scan by tiling-deletion and sequencing (CREST-seq) by using high-throughput CRISPR-Cas9-mediated mutagenesis (14) and discovered a large number of enhancer-like promoters regulating *POU5F1* expression. His lab recently further confirmed the genome-wide presence of enhancer-like promoters from 27 human cell/tissue types (15). Future work is required to systemically investigate the contribution of enhancer-like promoter in carcinogenesis and whether they have unique properties compared to canonical enhancers in cancer-specific gene regulation.

### Transposable elements

Transposable elements (TEs) are highly repetitive elements which make up 50% of the human genome (16). Although most TEs have lost their ability to transpose due to mutation and truncations, they encode functional promoters and enhancers, while harboring transcription factor binding sites and epigenetic regulatory signals to rewire the regulatory network. The essential role of TEs in driving cell-specific transcription in pluripotent cells has been documented (16). In most cases, TEs are silenced in somatic cells. DNA modifications (e.g. hypomethylation) or genetic variation (e.g. mutations and insertions) can result in gain or loss of transcription factor binding sites in TEs, which lead to transcriptional dysregulation in carcinogenesis (16). In a recent study, Ting Wang lab systemically analysed 7769 tumours and 625 normal datasets from 15 cancer types and identified 129 activated TE cryptic promoters to regulate the transcription of 106 oncogenes in 3864 tumours. They found that the presence of TEs in tumours are largely variant in different cancer types (e.g. 12% for glioma and 87% for squamous cell lung cancer), implying their different function in various cancers (17). Moreover, some of the most prevalent onco-exaptation (i.e. epigenetic derepression of TEs in cancer) candidates were associated with worse survival. For instance, AluJb can function as an alternative promoter to enhance LIN28B expression and form a fusion transcript to promote tumour proliferation and invasion. In addition, reactivation of AluJb occurs due to DNA hypomethylation, raising the possibility for using targeted DNA methylation approaches (e.g. dCas9-DNMT) in the treatment of TE-driven cancers (17).

## MEASURING THE REGULATORY EPIGENOME IN CANCER

Surveying the exposed DNA regions accessible to transcription factors and their cognate’s binding is an essential step to determine the activity of enhancers and promoters. DNase-seq and ATAC-seq (Assay for Transposase-Accessible Chromatin using sequencing) are the two most common methods to measure chromatin accessibility. DNase-seq uses DNase to digest DNA whereas nucleosome-bound DNA is protected. The digested fragment is then subjected to next generation sequencing to examine open chromatin (50 million uniquely mapping reads) and transcription factor footprinting (150–200 million uniquely mapping reads) according to ENCODE standards (https://www.encodeproject.org/data-standards/dnase-seq/). ATAC-seq uses mutated Tn5 transposase preloaded with sequencing adaptors and can simultaneously tag the accessible DNA with sequencing adaptors (called tagmentation), followed by PCR amplification and sequencing (18). ATAC-seq is a simple, bench-efficient method taking a few hours and only requires fresh 50,000 (or even 500) cells, enabling its wide applicability in chromatin accessibility mapping especially for samples with low cell number.

Chang and Greenleaf labs have further optimized their original ATAC-seq protocol and developed Omni-ATAC-seq (19), which substantially reduced the mitochondrial DNA contamination by introducing digitonin in lysis buffer. Moreover, this protocol enabled the interrogation of frozen tissues. As such, they mapped the chromatin accessibility in 410 biobanked primary tumour tissues obtained from The Cancer Genome Atlas (TCGA) representing 23 different types of human cancers. Those samples were performed for whole genome sequencing and RNA-seq, enabling multi-omics data integration to interrogate how the interplay of genetic variation and regulome drives carcinogenesis. In addition, these are great reference regulome maps for cancer research because chromatin accessibility mapping has also been done for K562 (leukemia) and GM12878 (lymphoblastoid), HepG2 (hepatocellular carcinoma), HeLa-S3 (cervial carcinoma), MCF-7 (breast cancer), A549 (lung adenocarcinoma) and SK-N-SH (neuroblastoma) cancer cell lines in ENCODE consortium (20), which complement data from primary tumours. In the future, large regulome mapping to understand the multilayered transcriptional regulation of each type of cancer comprising hundreds to thousands of samples can be performed to identify novel subtypes of cancers. Integrating clinical data for patients with such multi-omics data would further uncover novel biomarkers associated with disease prognosis and guide the clinical strategy for cancer patients.

Recently, single cell ATAC-seq (scATAC-seq) was developed independently by William Greenleaf lab and Jay Shendure lab, enabling the identification of the open/accessible chromatin at single cell level (21,22). After that, Eileen Furlong lab and Jay Shendure lab further collaborated to adapt the scATAC-seq protocol to work with nuclei from formaldehyde-fixed tissues (23). Based on scATAC-seq approach, it is possible to identify cell subpopulations with different chromatin accessibility profiles within complex samples. For instance, scATAC-seq approach has been employed to interrogate the formation of chromatin accessibility in human hematopoiesis (24,25), human acute myeloid leukemia (AML) (25), embryonic development in Drosophila (23) and mouse cell atlas (26). By combining the Chromium platform (10× Genomics), a massive scale of single cell mapping can be achieved. This has been used in tumour biopsies with pre- and post- PD-1 inhibition that identified therapy responsive-T cell subtypes and the associated regulatory programs in a report by Howard Chang, William Greenleaf and Grace Zheng laboratories (27). In addition, William Greenleaf lab has performed scATAC-seq and CITE-seq (cellular indexing of transcriptomes and epitopes by sequencing) profiling of mixed-phenotype acute leukemias (MPAL), showing that RUNX1 is a key transcription factor regulating the transcriptional signature in MPAL (28).

In the future, it will be attractive to apply this approach for decoding the regulome of various human cancers including tumour cell heterogeneity and their microenvironments (e.g. immune cells and tumour-associated fibroblast) in cancer development and progression. It will also allow analysing pre- and post-treatment with candidate compounds, providing novel insight into evolutional trajectories for cancers as well as identifying the unique subpopulations that are implicated in therapeutic resistance. Moreover, applying this approach on circulating tumour cells (CTCs) even allows tracking the alteration of cancer cells in ‘real time’ without invasive biopsy and assess the therapeutic response/outcome. Although scATAC-seq method is appealing, some technical challenges need to be overcome in the future: (i) its sensitivity to tissue dissociation and how to reduce the Tagmentation bias created by different cells subjected to the same nuclear lysis conditions; (ii) some computational methods (e.g. label transfer (26)) have been developed to integrate scATAC-seq and single-cell RNA-seq (scRNA-seq) data but pairing of subpopulations of scATAC-seq and scRNA-seq is challenging; (ii) the coverage for each single cell is still low due to capturing a small subset of open chromatin in one cell, and it is also computationally challenging to distinguish the uncaptured regions due to technical limitations and transcriptionally inactive regions. Hence, simultaneous measurements of chromatin accessibility and gene transcription in the same individual cell will be the ideal solution to solve this issue. In light of this, single-cell combinatorial indexing for jointly profiling of chromatin accessibility and mRNA (sci-CAR) has been developed (29). To confirm that the profiling is truly from the same cells, the authors mixed human and mouse cells and found that 99% of co-assayed cells were assigned to the same species, indicating its high accuracy to perform these two assays within the same cells.

It should be noted that chromatin accessibility alone is not sufficient to alter the activity of regulatory elements. A recent study revealed that during the progression of pancreatic cancer, enhancers are extensively reprogrammed but metastasis-specific enhancers have equivalent ATAC-seq signal compared to primary tumours, indicating the chromatin accessibility for those sites is pre-established in primary tumour (30). In addition, in T cells, most of subtype-specific enhancer loops shared equivalent chromatin accessibility on the anchors (31). This indicated that other parameters should be included to comprehensively evaluate the regulatory potential of the elements, for example using ChIP-seq for mapping active enhancer marks (e.g. H3K27ac). Traditionally, ChIP-seq requires millions of cells that limit its application in primary cancer samples. Recently, Cleavage Under Targets and Tagmentation (CUT&Tag) (32) and CoBATCH (33) were developed as novel low cell number ChIP-seq approaches by requiring as few as 60 cells. Moreover, their basic protocols can be further adapted for single cell analysis. These two approaches used the same principle: (i) nuclear permeabilization similar to ATAC-seq; (ii) incubating with primary antibody to bind the protein of interest (e.g. H3K27ac) in the nuclei; (iii) secondary antibody incubation that binds to primary antibody; (iv) addition of transposome consisting of protein A/G-Tn5 transposase (pA/G-Tn5) fusion protein loaded with sequencing adapters; (v) activation of Tn5 by adding Mg^++^ for *in situ* cutting of DNA/protein complex; (vi) PCR amplification. These two approaches dramatically reduce the required materials such as cells/tissues for ChIP-seq, increase signal–noise ratio, reduce sequence depth (about 10-fold) and can generate the sequence-ready libraries in the same day.

Although H3K27ac is the best marker for active enhancer, whether H3K27ac only correlates or is causal to enhancer activity, is a hotly debated question in the epigenetic field. To address this question, Bing Zhu lab made a point mutation of lysine 27 to arginine (an unmodifiable residue) on histone H3.3 (H3.3K27R) in mouse embryonic stem cells, which made the cells lose the capability of being acetylated. However, loss of H3K27ac at enhancer regions did not affect the chromatin accessibility, transcription and self-renewal of mouse embryonic stem cells (mESCs) (34). A previous study indicated that acetylation can neutralize positive charges on the lysine of histone, reduce the binding of histone and DNA and open the chromatin (35). However, this study suggested that in mESCs, single lysine residue acetylation alone is not sufficient to affect the enhancer activity and it might need to synergistically work with other lysine residue acetylations to make the chromatin loosen and maintain the active state of enhancers. The effect of H3K27ac on the enhancer activity of other cell types such as cancer cells would still need to be examined by using the same approach.

It should also be emphasized that assessing the functionality of enhancers only based on epigenomic mapping might not be sufficient due to the presence of redundancy: one enhancer can compensate the inactivation of another enhancer to regulate the same gene, which is supposed to confer the robustness in transcriptional activity (36,37) by buffering the genetic or epigenetic changes of individual enhancers. CRISPR-based *in vivo* genome-editing methods should be introduced to mutate or delete an enhancer, measure the resulting transcriptional output and thus confirm their functional contribution to transcription in cancer.

## ENHANCER–PROMOTER INTERACTION: A DRIVER OR PASSENGER FOR GENE ACTIVATION IN CANCER

**Figure 1.**
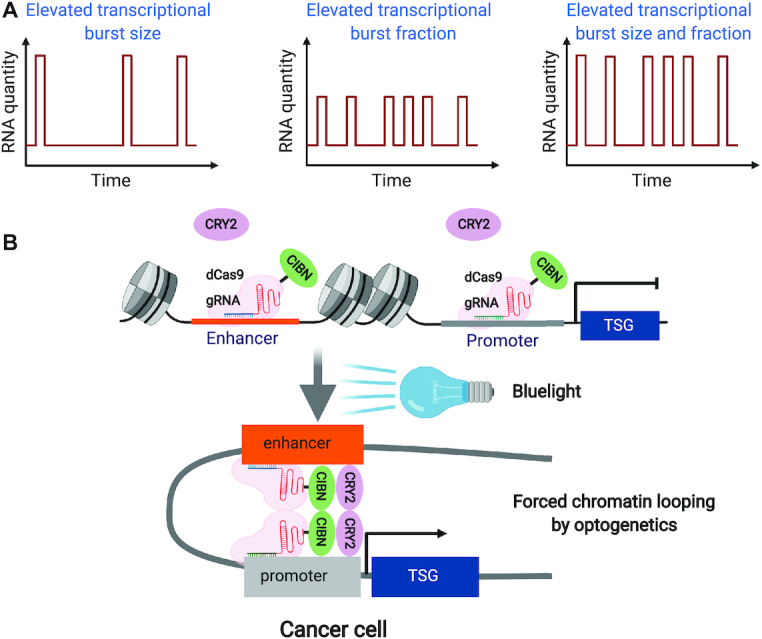
Manipulation of enhancer–promoter interactions in cancer. (**A**) Schematic models of how transcriptional burst can be modulated during active transcription. (**B**) A hypothetic example of using light-activated dynamic looping (LADL) technology to achieve optogenetic manipulation of the enhancer–promoter interaction of a tumour-suppressor gene (TSG) in cancer cell: guide RNAs (gRNAs) enable the localization of fusion protein (dCas9-CIBN) onto the targeted enhancer and promoter. Upon blue light stimulation, CRY2 protein heterodimerizes CIBN and juxtaposes enhancer and promoter to form the chromatin loop and induce transcription activation in cancer cells.

Many observations have indicated that the physical contact of enhancer–promoter is concomitantly changed with transcription. For example, during mouse erythroid differentiation, enhancer–promoter interactions were established concomitantly established with progressive upregulation of gene activity (38). However, whether enhancer–promoter interaction is a driver or just a passenger for active transcription is a key question in enhancer research. There is considerable evidence that supports the first notion. Pioneering studies from Blobel lab showed that forced tethering of enhancer to β-globin promoter by artificial zinc finger is sufficient to activate β-globin transcription in erythroblasts even in the absence of transcriptional activator GATA1 (39). Moreover, forced chromatin looping can even reactivate silenced fetal globin genes in adult erythroid cells (40). His lab further examined its underlying mechanism and found that forced enhancer–promoter interactions led to higher transcriptional bursting fraction (more events per time frame) but not higher bursting size (more RNA molecules per transcriptional event) (41) (Figure 1A), providing mechanistic insights on how enhancer–promoter interactions activate gene transcription. Besides, new approaches for forced enhancer–promoter looping using dCas9 have also been developed (42,43). However, these approaches to induce chromatin looping require relatively long time, which hampers the investigation of chromatin looping dynamics and its effects on transcriptional activation. Recently, light-activated-dynamic-looping (LADL) system was developed for inducible long-range interactions in response to bluelight (44). The LADL anchor is formed by fusing the architectural protein CIB1 and dCas9. The gRNA plasmid contains two functions: (i) produce the gRNAs to guide the LADL anchors to the two targeted loci and (ii) produce CRY2 protein to heterodimerize CIBN in response to bluelight and thus bridge the two anchors to form the chromatin loop. This powerful optogenetic approach has enabled faster and reversible chromatin looping engineering as compared to the previous approaches and can be harnessed for control of enhancer–promoter rewiring in temporal precision. Collectively, by using these tools, it is possible to manipulate the enhancer–promoter looping to investigate their roles in transcription regulation and functions in cancer cells (Figure 1B).

Compared to intrachromosomal chromatin interactions, interchromosomal chromatin interactions are highly controversial in the 3D genome field. One of the most contentious cases would be estrogen receptor (ER)-mediated chromatin interaction. For example, Michael Rosenfeld lab and Xiang-Dong Fu lab reported that estrogen could induce ERα-dependent interchromosomal interactions in the MCF7 breast cancer cell line (45). On the contrary, in the same cell line model, by using Chromatin Interaction Analysis with Paired-End Tag (ChIA-PET) and validation assays (3C and FISH), Yijun Ruan lab found that ERα-bound intrachromosomal interactions are bona fide, while the putative interchromosomal interactions are mostly false positive. Such equivocal findings have led to interchromosomal interactions being regarded as noise and filtered during sequencing data processing in most cases of 3D genome analysis.

However, a growing body of evidence also suggests the existence of interchromosomal interactions and their roles in physiological or pathological gene regulation (46). An example comes from Stavros Lomvardas lab, which demonstrated that extensive interchromosomal interactions were established during olfactory sensory neuron (OSN) differentiation. His lab showed that multichromosomal super-enhancers come into proximity and form a regulatory hub with a strong enrichment of transcription factor LHX2 and adaptor protein LDB1 to interact with the olfactory receptor (OR) gene promoters. Knockout of LHX2 or LDB1 led to dramatically disrupted interchromosomal interactions and decreased transcription of *OR* genes, suggesting the essential roles of interchromosomal interactions in transcriptional regulation of the olfactory system (47). Currently, interchromosomal interactions are still much less studied compared to intrachromosomal interactions. Future efforts to examine the landscapes and functions of interchromosomal interactions in diverse normal and human cancer cells might unravel whether they are rare phenomena or common features of human chromosomes in dictating cell-specific transcription. This will be particularly relevant for understanding transcriptional regulation during carcinogenesis.

Although strong evidence has revealed that proximity of enhancers and promoters may be a driver of transcription, in some cases, enhancer–promoter interaction alone might not be sufficient to drive transcription. In a recent study performing Capture-C approach at over 60 loci to test the impact of BET inhibitors on enhancer–promoter interactions and transcriptional change in an acute lymphoblastic leukemia cell line, the authors found that BET inhibition has a strong effect on transcription but enhancer–promoter interactions remained undisrupted (48). In addition, upon glucocorticoid treatment, glucocorticoid receptor (GR) did not reorganize chromatin looping but bound on the sites with pre-established enhancer–promoter interactions and modulated gene transcription (49). These studies suggested that the contribution of enhancer–promoter interaction to transcription activation is not uniform and chromatin-context dependent.

Furthermore, enhancer modulated gene expression might not need direct contact with promoters. For example, rs9349379 is a causal SNP of five vascular diseases by modulation of *EDN1* expression (50). rs9349379 resides in an enhancer about 600 kb away of *END1* gene and modulates the enhancer activity marked by H3K27ac. Only minimal contact of rs9349379 and *EDN1* promoter was detected, indicating that the effect of rs9349379 on *EDN1* gene expression is not through traditional enhancer–promoter interaction. Interestingly, both sites have strong interaction with a common enhancer about 300 kb away from each site. Further work is needed to unravel whether rs9349379 can indirectly modulate EDN1 interaction through this common site (50). In addition, in mouse ESC, the essential enhancer for *Sox2* activation has no enhanced spatial proximity to *Sox2* promoter (51). Moreover, Wendy Bickmore lab revealed that for enhancer-driven activation of the Sonic hedgehog gene (Shh) in mouse ESC, the enhancer has a decreased juxtaposition with *Shh* promoter (52). Although it cannot be excluded that the existence of enhancer–promoter communications are too transient and cannot be captured by current techniques, these studies suggest that the function of enhancers in transcription regulation might not only be mediated through a simple enhancer–promoter looping.

## NEW PLAYERS IN ENHANCER–PROMOTER INTERACTIONS

### Enhancer RNAs (eRNAs)

A decade ago, Gioacchino Natoli lab and Michael Greenberg lab independently reported that active enhancers can give rise to transcription (53,54), and the products are termed enhancer RNAs (eRNAs). Due to their short half-lives compared to mRNA and lncRNAs (55), annotations of eRNAs depend on nascent RNA sequencing approaches, such as Cap Analysis of Gene Expression (CAGE) (56,57)/native elongating transcript–cap analysis of gene expression (NET-CAGE) (58), Global Run-On Sequencing (GRO-Seq) (59) or its refinement Precision Run-On Sequencing (PRO-seq) (60). Although pervasively observed, it was first unclear, whether eRNAs are key players in transcription or merely a by-product of enhancer activation In recent years, growing evidence has suggested that eRNAs are important in transcriptional activation through modulation of chromatin accessibility (61), augmentation of histone acetylation via interaction with histone acetyltransferase CBP (62) or stabilization of enhancer-promoter looping (63,64). RNA *in situ* conformation sequencing (RIC-seq) for analysing global RNA–RNA interactions has further revealed that the connectivity of enhancers and promoters can be assigned using their pairwise-interacting RNAs (65). Interestingly, the authors found that the super-enhancer *CCAT1–5L* derived eRNA (*CCAT1–5L* SE RNA) binds *MYC* promoter and eRNA, and physically interacts with hnRNPK, whose oligomerization facilitates enhancer–promoter looping, RNAPII occupancy and transcription of *MYC*.

### Polycomb group proteins

Historically, polycomb group proteins (polycomb repressive complexes 1 and 2 (PRC1 and PRC2)) are considered as transcriptional repressors. However, increasing evidence has shown that they can function also in transcriptional activation. For example, PRC1 can bind on active enhancers and promoters devoid of repressive histone mark H3K27me3 (66–69). Cavalli lab showed that a subset of PRC1 binding is associated with increased enhancer–promoter interactions, which are dynamically altered and contributes to transcriptional activation during development in *Drosophila* (70). A recent study identified PRC1 as a driver in prostate cancer metastasis (71). EZH2 (the catalytic component of PRC2) can also function as a transcriptional activator independently of methylation activity in driving prostate cancer development (72). Whether PRC1 and PRC2 drive carcinogenesis, at least in part, through activating enhancer–promoter interactions but not canonical repressor function needs further investigation. Moreover, polycomb-bound transcriptional silencers can switch to transcriptional activators during development (73), while systemic identification of transcriptional silencers in cancers is limited. Whether and how they switch into transcriptional activators and reorganize the chromatin interactome during cancer development and progression remains to be further studied.

### Lamins

The inner nuclear membrane is lined by the nuclear lamina which consist of lamins. Parts of chromatin interact with the lamina and form lamina-associated domains (LADs) (74). Most of the genes in LADs are transcriptionally inactive, suggesting that lamins function as transcriptional repressors. Besides the inner nuclear membrane, lamins also reside in nuclear interior. A recent study reported that phosphorylated lamin A/C bound on a subset of putative enhancers that demarcated with accessible chromatin and active enhancer mark (H3K27ac) in the fibroblast of Hutchinson–Gilford Progeria Syndrome (HGPS) patients. Those enhancers were linked to transcriptional activation of genes implicated for the phenotypes in HGPS patients and resided in inter-LADs but not intra-LADs regions. However, non-phosphorylated lamin A/C did not bind on those enhancers and resided in intra-LADs (75). How phosphorylated lamin A/C is recruited onto active enhancers and how phosphorylated lamin A/C regulate enhancer activity warrant further studies.

### Extrachromosomal DNA (ecDNA)

Another new player for enhancer–promoter interactions is extrachromosomal DNA (ecDNA), which was first visualized in neuroblastoma in 1965 (76,77). A comprehensive analysis found that about half of human cancers have ecDNA (78), implying their functional significance in cancer development. In light of this, ecDNA has been found to be circular and drive massive expression of oncogenes in ecDNA. In addition, the high expression of oncogenes in ecDNA did not solely depend on the increased copy number of oncogenes but harbors highly accessible chromatin with active histone marks that enables their frequent long-range interactions with oncogene promoters (79). In line with this, the authors found that in multiple solid tumours, most of ecDNA contain enhancers, and they are co-selected with the oncogenes during the formation of ecDNA (80). These enhancers are presumed to be derived from tumour cell chromosomes but can ‘escape’ from the chromosomes.

## ALTERATION OF CHROMATIN TOPOLOGY IN CARCINOGENESIS

**Figure 2.**
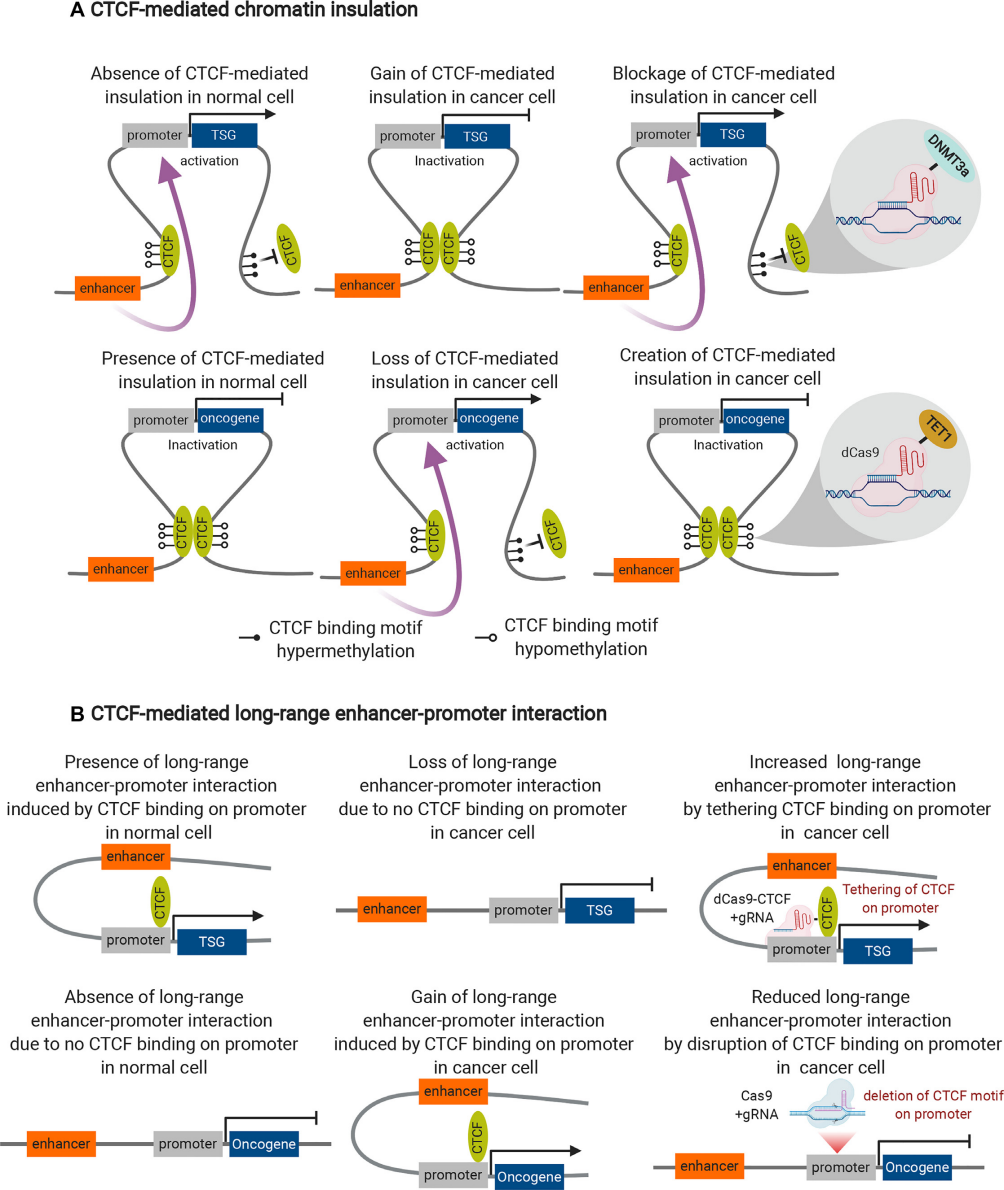
Control of CTCF-mediated chromatin topology in cancer. (**A**) CTCF-mediated chromatin insulation: in a normal cell, DNA hypermethylation disrupts CTCF binding and looping, leading to abrogation of chromatin insulation, gain of enhancer–promoter interaction and transcription activation of tumour suppressor gene (TSG) or transcription repression of an oncogene. In a cancer cell, DNA hypomethylation facilitates CTCF binding and looping, leading to establishment of chromatin insulation, loss of enhancer–promoter interaction and transcription inactivation of tumour suppressor gene (TSG) or transcription activation of an oncogene. Epigenetic editing of CTCF-binding sites on the insulation anchors for TSG (dCas9-DNMT3a) and oncogene (dCas9-TET1) can lead to blockage or creation of CTCF insulation, gain or loss of enhancer–promoter interaction and transcription activation or inactivation for TSG and oncogene, respectively, in cancer cell. (**B**) CTCF-mediated long-range enhancer–promoter interaction: in a normal cell, CTCF binding on TSG promoter leads to establishment of enhancer–promoter interaction and transcription activation of TSG (vice versa for oncogene). In a cancer cell, loss of CTCF binding on TSG promoter leads to abrogation of enhancer–promoter interaction and transcription inactivation of TSG (vice versa for oncogene). Artificial tethering of CTCF on the TSG promoter via dCas9-gRNA approach can re-establish the enhancer–promoter interaction and transcription activation of TSG in cancer cell. Deletion of CTCF motif via Cas9-gRNA approach disrupts CTCF binding on the oncogene promoter, leading to abrogation of enhancer–promoter interaction and transcription inactivation of oncogene in cancer cells.

In most cases, alteration of 3D genome organization occurs at the level of chromatin loops. For instance, insulated neighbourhoods are defined by chromatin loops formed by CTCF–CTCF homodimer (81) and are the major structuring components of TADs whose boundaries are demarcated by strong CTCF binding (82). The CTCF-enriched boundaries can serve as insulators to confine the enhancer–promoter interactions within Insulated neighbourhoods/TADs. The loss of Insulated neighbourhoods or TADs boundary has been well documented in carcinogenesis, which arise from structural variation or loss of CTCF binding due to DNA methylation (4). For example, microdeletion eliminates the boundary of Insulated neighbourhoods in T-cell acute lymphoblastic leukemia (T-ALL), which is linked to the proto-oncogene *TAL1* activation (83). DNA hypermethylation impaired CTCF binding on the loop anchors and disrupted the insulation that partitions an enhancer from *PDGFRA* oncogene in glioma (84), a super-enhancer from *FGF4* oncogene in gastrointestinal stromal tumours (GISTs) (85), which create aberrant enhancer–promoter interactions and gene activation. The contributions of chromatin insulation in cancer development can be further explored via modulation of DNA methylation on CTCF-bound insulator using dCas9-DNMT3a (DNA methylase) or dCas9-TET1 (DNA demethylase) (Figure 2A).

By Chromatin Interaction Analysis with Paired-End Tag (ChIA-PET) mapping in a B-lymphocyte cell line, Yijun Ruan lab found that the CTCF loop structures can be divided into ‘anchor’ and ‘loop’ regions (i.e. the loops covering the genes/enhancers). Anchor regions are enriched for active epigenomic marks, RNAPII binding and transcription start sites (TSSs), suggesting that CTCF can function as foci for transcriptional factories (86). In line with this, Bing Ren lab recently revealed that disruption of CTCF binding on the promoter led to reduction of promoter–enhancer interaction and decreased transcription, while artificial tethering of CTCF on the promoter led to restoration of enhancer–promoter interaction and transcription. Moreover, the occupancy of CTCF on the promoter is associated with cell-specific transcription (87). In addition, Keji Zhao lab uncovered that CTCF could bring distal enhancer to the vicinity to their target genes via interaction with cohesin (88). These studies suggested the distinct role of CTCF in promoting enhancer–promoter interactions beyond its insulation function. Thus, a broader survey will be needed to determine the extent to which cell-specific CTCF binding on the promoter/enhancer dictates cell-specific transcription in cancers (Figure 2B). In addition to CTCF, transcription factor YY1 can induce enhancer–promoter interactions in mESC by binding on hypomethylated DNA and form YY1 homodimer (89). Whether YY1 is overexpressed in a wide-range of cancers, and whether YY1-mediated enhancer–promoter interactions are universal features in cancer cells needs further investigation.

Moreover, recent study indicated that Brother of The Regulator of Imprinted Sites (BORIS, also known as CTCFL), a paralog of CTCF, is aberrantly upregulated in prostate, ovarian, lung and breast cancers (90–93) and its expression is correlated with tumour characteristics and therapeutic response (94). By HiChIP targeting SMC1A (a subunit of cohesin) in ALK inhibitor sensitive versus resistant neuroblastoma cancer cells, the authors found that most of gained chromatin loops in resistant cells have strong enrichment of BORIS on the anchors while the binding of CTCF on those anchors are equivalent (94). Moreover, knockdown of *BORIS* led to reduced SMC1a binding and more than a quarter of loops were lost, supporting loop extrusion model (95) and the roles of BORIS in regulating chromatin looping. It will be interesting to further validate these findings in the patient samples during the evolution of resistance and further examine whether ectopic chromatin topology drive target therapy/chemotherapy/immunotherapy resistance in a broad range of cancers.

A recent report showed that the lncRNA *PVT1* promoter competed with the enhancers of its neighbour gene *MYC* (96), and thus reduced promoter–enhancer looping for *MYC* and decreased *MYC* expression. In contrast, in malignant cells, *PVT1* promoter was inactive due to epigenetic silencing or structural variation, and thus restored the enhancer–promoter looping for *MYC*, thereby increasing *MYC* expression and tumour growth.

Although the authors proposed *PVT1* promoter as a novel insulator-like element, promoter competition could still account for these effects, as observed by Peter Fraser lab (97), Douglas Higgs lab (98) and Gerd Blobel lab (41) at the globin loci. Relocalizing the *PVT1* promoter to the other side of the enhancer and examining whether the effect is lost would help to clarify this aspect. If it is an insulator, future work is also needed to identify the epigenetic markers that distinguish the insulator-like promoters as compared to classical promoters and how the promoter insulation and TAD insulation are differed from each other and coordinate for cancer-specific gene expression.

## PHASE SEPARATION: A NEW REGULATOR IN CHROMATIN ARCHITECTURE

Compartmentalization is crucial to create functionally distinct units within a cell for diverse biochemical reactions with different concentration of certain factors (e.g. proteins) in the compartment. Besides the lipid bilayer membrane that defines the boundary of compartments, membrane-less compartments can be formed through a process called liquid–liquid phase separation (LLPS), a process characterized by liquid droplets phase separating from the aqueous environment. Defects in LLPS have been linked to carcinogenesis. A recent study demonstrated that Speckle-type POZ protein (SPOP), the substrate-binding subunit of a cullin 3 (CUL3)-based E3 ubiquitin ligase complex, is triggered by substrate (proto-oncogenic protein) binding to form phase separation (nuclear membraneless organelles) for the ubiquitination and subsequent proteasomal degradation of proto-oncogenic proteins. However, mutation of SPOP, which is frequently observed in prostate cancer and other cancers, disrupted SPOP–substrate co-localization and phase separation, thus reducing ubiquitylation and proteasomal degradation of proto-oncogenic proteins (99).

In addition, recent explosion of research on LLPS has led to a series of discoveries on chromatin domains and enhancer regulation. For instance, phase-separated heterochromatin protein 1 (HP1) droplets mediated chromatin compaction and the formation of heterochromatin domain (100,101). Moreover, Richard Young lab showed transcription factor co-activator BRD4, MED1 form nuclear condensates and those droplets created compartmentalization and distinct local high-concentration environments that facilitate the biochemical reactions for transcription (102). His lab further found that phase separation of activation domains is a general property of TFs (103).

Although intrinsically disordered regions (IDRs) in transcription factors are considered as a general mechanism for LLPS, the new roles of RNA in regulation of LLPS are being explored (104). The first report came from Rosenfeld lab showing that treatment of MCF7 breast cancer cells with estrogen induced the assembly of enhancer RNA (eRNA)-dependent ribonucleoproteins (eRNPs) complex with condensins and these eRNPs are essential for the formation of phase separation on enhancer and its function, suggesting that eRNAs can function as a scaffold and mediate the formation of a phase-separated enhancer structure (105). It was revealed that localization of YY1 on enhancers and promoters depends on its binding with RNA (106), which underlie the potential role of their interaction in YY1-mediated enhancer-promoter looping. In addition, an eRNA transcribed from mouse Chromosome 7 mediated the recruitment of SMC3 (a subunit of cohesin) on *Myogenin* located on Chromosome 1, leading to transcriptional activation of *Myogenin* (107). In line with this, two recent approaches termed global RNA interactions with DNA by deep sequencing (GRID-seq) (108) and chromatin-associated RNA sequencing (ChAR-seq) (109) identified that chromatin-associated RNAs are largely involved in long-range chromatin interactions. Danny Reinberg lab identified the RNA binding regions in CTCF, and their mutation led to markedly decreased chromatin looping and altered transcription, suggesting that CTCF-mediated genome organization is orchestrated by RNA interaction (110). Inspired by these studies, we propose that future studies should investigate whether the impact of CTCF/YY1 and RNA interaction on chromatin topology is ultimately regulated by RNA-mediated LLPS, and to further unravel what are the key determinants of RNA-mediated LLPS (RNA modification, RNA sequence, secondary structure etc.) in chromatin structure? Addressing these questions will help to have a better understanding of the mechanisms underlying aberrant chromatin architectures in cancers. Moreover, by blocking the formation of such aberrant condensates with specific strategies may lead to new therapeutic approaches for cancers by reversing chromatin architectures to their physiologically normal state.

## NOVEL APPROACHES FOR 3D GENOME MAPPING

Current Hi-C (in solution and *in situ* Hi-C) approaches are able to capture all-to-all interactions in the nucleus but are required to generate many replicates of libraries for ultra-deep sequencing (111). This requires much material for producing libraries and involves considerable costs for sequencing. Particularly, it is unrealistic for primary cancer samples. Thus, in many current publications, the sequencing depth is not enough to examine enhancer–promoter interactions.

Amos Tanay lab developed a novel 4C approach, called unique molecular identifier (UMI)-4C, which can examine the chromatin interactions for one bait or multiplexed baits (up to 20–50) at high resolution (112). In addition, Douglas Higgs/Jim Hughes labs further refined their previously developed Capture-C approach (113) and presented a new next-generation (NG) Capture-C protocol (114). By using a new oligonucleotide capture process, NG Capture-C mapping achieved unprecedented sensitivity and resolution of mammalian genomes. The initial Capture-C protocol required the design of a minimum of 40 000 probes (irrespective of the number of desired viewpoints). However, in NG Capture-C protocol, each set of capture oligos only targets one to many hundreds of regions and new capture oligos can be added to the existing pool for the experimental need (114), thus providing great flexibility and dramatically reducing the cost for an experiment with a small design.

**Figure 3.**
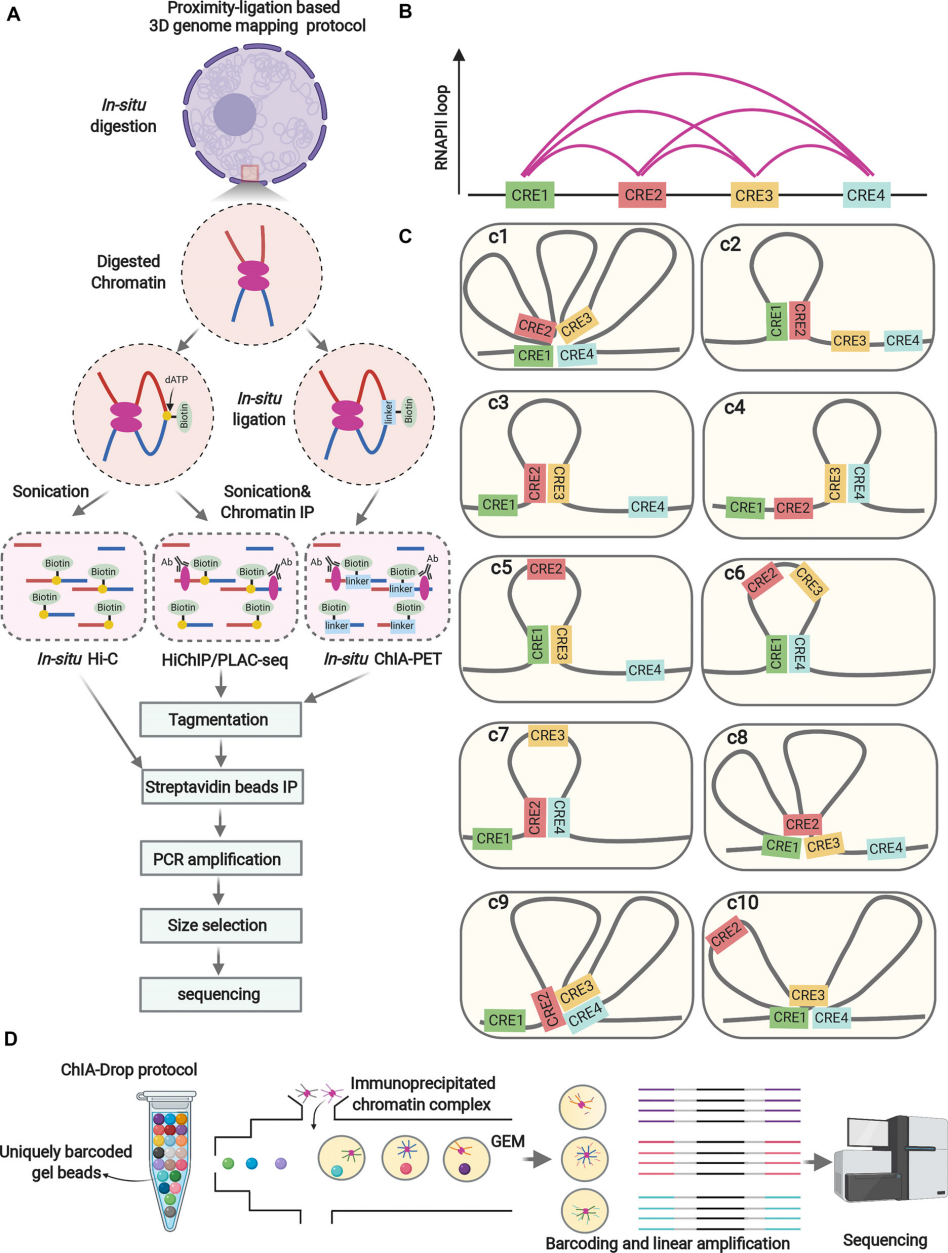
Dissection of 3D chromatin architecture in cancer. (**A**) Proximity-ligation based 3D genome mapping protocol: crosslinked cells are subjected to nuclei isolation. *In situ* enzymatic digestion of chromatin is performed, followed by *in situ* ligation (*in situ* Hi-C and HiChIP/PLAC-seq: biotin-labeled dATP, *in situ* ChIA-PET: bio-labeled bridge linker) of two chromatin fragments that are proximal in space. Thereafter, the nuclei are subjected to sonication. For HiChIP/PLAC-seq and *in situ* ChIA-PET, chromatin immunoprecipitation is required to capture the specific protein-centric chromatin interactions (e.g. RNAPII). Subsequently, the chromatin for all these assays is treated by de-crosslinking and DNA purification. Then the purified DNA is tagmented by transposon (Tn5) preloaded with sequencing adapters, followed by streptavidin bead immunoprecipitation (to capture the DNA fragments conjugated with biotin), PCR amplification, size selection and sequencing. (**B**) Example of a chromatin interaction cluster (multiplex chromatin structure) that involves four *cis*-regulatory elements (CREs), as revealed by HiChIP/PLAC-seq or *in situ* ChIA-PET using RNAPII antibody. (**C**) Ten possible scenarios of interactions in individual cells speculated based on (B). Current proximity-ligation based 3D genome mapping technologies can only detect binary chromatin interactions, and cannot explore the existence of multiplex chromatin structures in single chromosome and its cellular heterogeneity, which hardly allow a clear view of the chromatin interactions and thus miss their implications in cancer. For example, it cannot be judged whether those chromatin interactions co-exist in individual cells (3c1) or are they just the ensemble of various chromatin interactions from individual cells (3c2-c10). (**D**) Illustration for the experimental procedures of ligation-free 3D genome mapping technology ChIA-Drop: crosslinked cells are subjected to sonication and chromatin immunoprecipitation. The precipitated chromatin is then loaded into the microfluidic device to generate gel-bead-in-emulsion (GEM). Each GEM from contains the reagents and unique barcodes for PCR amplification and sequencing. Amplicons from the same GEM have the same barcodes, enabling the inference of multiplex chromatin interactions.

In addition to Capture-C approach, protein-centric approaches (e.g. HiChIP (115) and Proximity Ligation-Assisted ChIP-seq (PLAC–seq) (116), *in situ* ChIA–PET (117)) using an antibody of interest (e.g. H3K27ac, RNAPII, CTCF and SMC1a), can also examine chromatin looping at high-resolution with dramatically reduced sequencing depths and starting materials (e.g. one million cells), providing the technical basis for 3D genome mapping in cancer research using primary cells/tissues. It had been observed that many regulatory elements such as enhancers and promoters could be aggregated into multiplex chromatin structures, suggesting this might be the topological basis for gene co-regulation. However, the aforementioned 3D genome technologies are based on proximity-ligation and paired-end-tag (PET) sequencing and detect only binary interactions (Figure 3A). In addition, the multiplex chromatin structure mapping results derived from those approaches were mixed results derived from millions of cells. Thus, it cannot reflect true multiplex chromatin interactions in single chromosomes and does not reflect chromatin heterogeneity among individual cells. These limitations have created the biases in visualizing the bona fide picture of chromatin organization (Figure 3B,C). Considering these biases, three ligation-free approaches ((genome architecture mapping) GAM (118), (split-pool recognition of interactions by tag extension) SPRITE (119) and chromatin-interaction analysis via droplet-based and barcode-linked sequencing (ChIA-Drop) (120)) were recently developed to examine genome-wide chromatin interactions. All these approaches can detect the genome-wide chromatin interactions between three or more genomic loci simultaneously. GAM uses ultrathin cryosectioning with a 220 nm thickness to dissect the nuclei into slices, and then for each slice, the DNA is extracted, barcoded and amplified before all the slices are multiplexed for sequencing. For example, 400 slices (each slice = one million sequencing reads) can achieve about 30 kb resolution. SPRITE and ChIA-Drop require crosslinking before chromatin preparation. These two methods need chromatin fragmentation (TurboDNase I for SPRITE, MboI for short chromatin fragment and sonication for long chromatin fragment (∼6 kb)) for ChIA-Drop) but do not require proximity ligation. For SPRITE, the fragmented chromatin is splitted into 96-well plate (each well has unique barcode), re-pooled and splitted into 96-well plate for couple rounds for barcoding and sequencing. For ChIA-Drop, the fragmented chromatin was subjected to chromatin immunoprecipitation to capture specific protein (e.g. RNAPII and CTCF) associated chromatin interactions. Then, the immunoprecipitated DNA was loaded onto the microfluidic device to generate gel-bead-in-emulsion (GEM) droplets. Each droplet contains unique barcode and the reagents for DNA amplifications (Figure 3D). Data analysis can be done by a newly developed pipeline called ChIA-Dropbox (121). By employing RNAPII ChIA-Drop in *Drosophila* S2, Ruan lab found that 80% of RNAPII-associated chromatin complex contains only one promoter. Although the remaining 20% of chromatin complexes contain multiple promoters and supported the notion of transcription factories (122), promoter–promoter interactions are less common than previously supposed (10). It would be interesting to perform ChIA-Drop only using the sonicated chromatin without chromatin immunoprecipitation and ultra-deep sequencing (billions of reads) to generate ‘all-to-all’ 3D contact map. This modification would potentially identify the chromatin interactions at single-molecule resolution, which are not associated with common histone marks (e.g. H3K27ac)/transcription factors (e.g. RNAPII) related to active transcription or architectural proteins (e.g. CTCF/Cohesin/YY1) related to chromatin topology. However, considering the sequencing cost, protein-centric ChIA-Drop would be a more ‘economic’ option for epigenome consortia (e.g. ENCODE, 4D Nucleome) to generate large-scale 3D chromatin maps at single-molecule resolution.

Visualization can further validate the findings from sequencing-based approaches and allow investigating the variability of individual cells. The most common method is 3D DNA FISH in fixed cells. For example, this approach was recently employed to visualize the 3D Cliques governing Type 1 diabetes in individual T cells (123). Stochastic Optical Reconstruction Microscopy (STORM) and Photoactivated Localization Microscopy (PAML) have been widely used for super-resolution imaging of 3D genome organization (124). For example, by using a modified STORM approach, Xiaowei Zhuang lab identified TAD-like domains at the single-cell level in the absence of cohesin (125). CRISPR/Cas9 system has been employed to tag genomic loci in live cells using guide RNA conjugating catalytically inactive forms of the Cas9 (dCas9) with green fluorescent protein (GFP), red fluorescent protein (RFP) or blue fluorescent protein (BFP) (126). A more robust approach termed Chimeric Array of gRNA Oligonucleotides (CARGO)-dCas9 was recently developed by delivering multiple RNAs to guide dCas9 and efficiently label the regulatory elements, and track their dynamics in living cells (127). With the improvement of imaging techniques, more genomic loci can be probed simultaneously providing more comprehensive views of how the genome is reorganized during the progression of carcinogenesis.

## CAN CHROMATIN TOPOLOGICAL STRUCTURES BE PRE-ESTABLISHED PRIOR TO TRANSCRIPTION DURING CANCER DEVELOPMENT AND PROGRESSION?

A recent report showed that there are two classes of enhancer–promoter interactions in progenitor and differentiating primary human epidermal keratinocytes (128). The enhancer–promoter interactions in Class I were mediated by differentiation-induced transcription factors with minimal cohesin binding and these were specific for differentiating primary human epidermal keratinocytes. Class II were the enhancer–promoter interactions pre-established in progenitor cells prior to gene expression. It is currently not known whether similar scenarios exist in tumorigenesis. It would be interesting to investigate the 3D genome trajectory during tumour development by investigating pre-cancerous lesions, primary tumour and metastasis to validate this hypothesis. For example, it is unclear whether enhancer–promoter interactions or the topological architectures are pre-established in earlier stages for genes that are essential for cancer development and progression, prior to their transcriptional changes.

**Figure 4.**
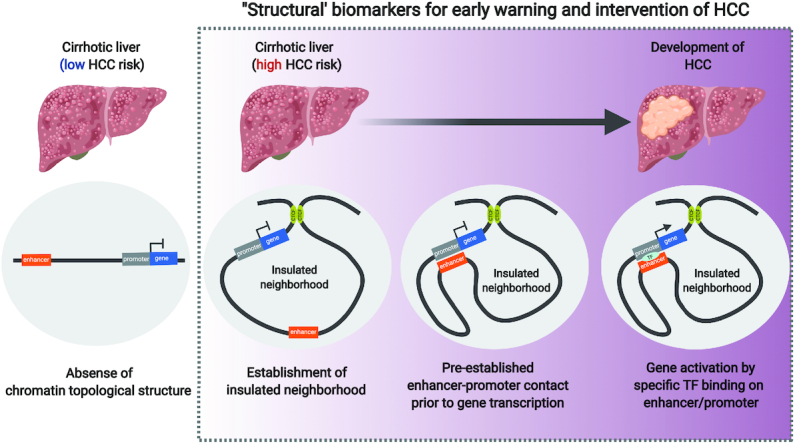
Pre-established chromatin topology in cancer development. Most of hepatocellular carcinoma (HCC) are developed on the basis of liver cirrhosis while the risk of HCC varies on cirrhotic patients. We presume that some cirrhotic patients are at low risk of HCC due to absence of chromatin topological structures orchestrating HCC development. Other cirrhotic patients are designated for HCC development due to establishment of the specific chromatin topological structures at stepwise fashions (i.e. establishment of insulated neighbourhoods, pre-established enhancer–promoter contact prior to gene transcription and gene activation by specific transcription factor binding on enhancer/promoter). Identification of these ‘structural’ biomarkers may be implicated for early warning and intervention of HCC development.

The Human Tumor Atlas Network (HTAN) as part of the National Cancer Institute (NCI) Cancer Moonshot Initiative was recently established and precancerous lesion is one of their major focus (129). We propose that high-resolution 3D genome mapping should be included in this project for decoding the chromatin topology in the precancerous stage. For example, hepatocellular carcinoma (HCC) is the fourth most common cause of cancer-related death worldwide, which results from chronic hepatitis (e.g. chronic hepatitis B) and subsequent precancerous stage (i.e. hepatic cirrhosis). Unfortunately, due to suboptimum for routine surveillance, >80% of HCC patients present at advanced stages and thus are unacceptable to receive curative surgical resection or transplantation, leading to dismal prognosis. Hence, discovering the biomarkers by 3D genome mapping for screening the patients designated for HCC development is valuable for early warning of HCC. We presume that some chromatin interactions (e.g. CTCF loops or enhancer–promoter interactions) may be pre-established, prior to gene transcription, in the cirrhotic patients with designated fate for HCC development. When specific transcription factors bind on the enhancer or promoter with pre-established contact, oncogene is transcriptionally activated, leading to the development of HCC (Figure 4). In future studies, liver biopsy followed by 3D DNA fluorescence *in situ* hybridization (FISH) can be applied to detect those ‘structural’ biomarkers in cirrhotic patients and screen the high-risk population for HCC development, who will be subject to intensive surveillance in regular follow-up care. In many cases, it is essential to use super-resolution microscope for 3D DNA FISH. However, for the enhancers which are far away from their promoters (e.g. ZRS enhancer, located 1 Mb from the *Shh* gene (130), and a *Myc* enhancer located 1.7 Mb downstream (131)), we might be able to use confocal microscope. Alternatively, it might be possible to use a panel of loci as selected markers and use locus-specific amplification methods (e.g 3C-qPCR) that would be technically feasible as an alternative for 3D DNA FISH. Moreover, based on aforementioned dCas9 approaches targeting CTCF looping or the tools targeting enhancer–promoter interactions, we could investigate whether manipulation of the 3D chromatin architectures in precancerous stage can halt the HCC development in animal model and this strategy could be applied to other cancer types.

## 3D GENOME ARCHITECTURE IN REPLICATION TIMING

DNA in mammalian cells is replicated in a defined temporal order in S phase known as replication timing (RT). Alteration of RT program occurs in units of replication domains (RDs) (400–800 kilobases) (132), which is highly correlated with A/B compartments (133,134). David Gilbert lab showed that the boundaries of some TADs aligned strongly with the boundaries of RDs, leading to the speculation about their relationship. However, in a recent study, his lab showed that deletion/inversion of TAD boundary or degradation of CTCF by auxin-inducible degron (AID) system in mouse ESCs (135) had no effect on RT (136), indicating that TAD boundaries and CTCF are dispensable for RT maintenance. Interestingly, his lab identified *cis*-regulatory elements called early replicating control elements (ERCEs) as the key regulators of RT. Deletion of ERCEs coincided with early-to-late RT shift, A-to-B compartment switch, disruption of TAD architecture and transcription inactivation. However, the deletion of some ERCEs led to significantly delayed RT but no changes in transcription, indicating that replication timing and transcriptional activity could be uncoupled at least in some cases. ERCEs were enriched for the hallmarks of active enhancers/promoters (DHSs, H3K27ac, H3K4me1, H3K4me3 or Med1) and transcription factors that control cell identity, suggesting ERCEs may function in a cell-type specific manner. Inversion of ERCEs was sufficient to create a new TAD boundary, suggesting ERCE is a novel 3D genome organizer. However, these elements were the anchors for CTCF-independent chromatin loops. In the future, it would be interesting to examine any previously unrecognized structural proteins (rather than CTCF/Cohesin/YY1) or ncRNAs that work in concert with ERCEs in 3D genome organization.

## CONCLUSIONS

Considerable progress has been made in recent years in identifying the genetic causes of cancers, such as the great efforts from the International Cancer Genome Consortium (ICGC) (https://icgc.org/) and The Cancer Genome Atlas (TCGA) (https://www.cancer.gov/about-nci/organization/ccg/research/structural-genomics/tcga). Many genetic variations are identified in cancers but mechanistically it is still poorly understood why genetic variation causes malignancy. This may be due in part to the current focus of research on the sequence but not the structure of the genome, particularly in the 3D view. In this perspective, we laid out the recent progress, arguments and our opinions of chromatin architecture in cancer development and progression. With technological and methodological advances, it will be possible to gain ever more insight to the dynamics of chromatin, including the regulatory hierarchies from nucleotide sequences to 3D chromatin architecture, as well as intratumoural cellular heterogeneity, metastasis and clonal evolution. We believe that by investigating cancer development and evolution through the lens of 3D chromatin architecture, it will be possible to uncover many new etiological mechanisms that have so far been elusive. Such studies will not only help understand the pathogenesis of cancer but also open new avenues for diagnostics and therapeutic interventions for cancer.
